# Correction

**DOI:** 10.1002/ueg2.12371

**Published:** 2023-03-07

In the article entitled “**The role of gut microbiome in inflammatory bowel disease diagnosis and prognosis**,”^1^ the funding information was missing. The following text should have appeared in the article:

This study was (partially) funded by InnoHK, The Government of Hong Kong, Special Administrative Region of the People's Republic of China, National Nature Science Foundation of China (82100573) and Croucher Senior Medical Research Fellowship.
